# A Meta-Analysis of Cigarette Smoking Prevalence among Adolescents in China: 1981–2010

**DOI:** 10.3390/ijerph120504617

**Published:** 2015-04-27

**Authors:** Juan Han, Xinguang Chen

**Affiliations:** 1 Department of Child and Adolescent and Maternal Health, School of Public Health, Tongji Medical College, Huazhong University of Science and Technology, 13 Hangkong Road, Wuhan 430032, China; 2 Pediatric Prevention Research Center, Wayne State University, Detroit, MI 48201, USA; 3 Department of Epidemiology, University of Florida, Gainesville, FL 32610, USA

**Keywords:** adolescents, tobacco use, meta-analysis, China

## Abstract

*Background*: Systematic data regarding adolescent smoking are needed at the national level to support evidence-based tobacco control in China. The goal of this study was to estimate smoking prevalence among Chinese adolescents using published data. *Methods*: Published studies were located electronically from the commonly used databases in Chinese and English, complemented by manual searching. Forty-five studies were selected of the 9771 retrieved from the databases. These studies targeted adolescents aged 12–17 or middle/high school students, were conducted during the 1981–2010, and had adequate data for meta-analysis. The 45 selected studies covered 52 sites in different parts of China. Smoking rates were estimated using the sample-weighted and random effect method. *Results*: The estimated prevalence rate of lifetime smoking (ever smoked) varied within a narrow range (39.04%–46.03%) for males and progressively increased from 2.47% in 1981–1985 to 19.72% in 2001–2005 for females. The prevalence rate of current (30-day) smoking for males declined from 26.62% in 1981–1985 to 10.86% in 1996–2000 before increasing again. The prevalence of current smoking for females increased from 0.29% in 1981–1985 to 3.26% in 2006–2010. *Conclusions*: The high levels of male smoking and the rapid increase in female smoking indicate growing burdens from tobacco-related diseases, underscoring the urgent need to strengthen adolescent tobacco control in China.

## 1. Introduction

Tobacco control in China presents a great public health challenge [1]. Accompanying the rapid economic growth in China is an increasing trend in tobacco related morbidity and mortality, including lung cancer and cardio-cerebrovascular diseases [2,3,4]. In addition to high rates of tobacco use among adults, more and more Chinese adolescents are now smoking. Data from the 2010 China Census indicated that the adolescent population 12–17 years of age totaled 104.11 million, accounting for 7.8% of the total Chinese population. Data from the 1999 Global Youth Tobacco Survey (GYTS) indicated that among youth, 16.2%–30.1% initiated smoking and 8.6-14.6% had smoked in the past month [5].

Adolescence represents a critical period in the life course. It is characterized by rapid and unevenly paced physical, psychosocial and neurocognitive development [6,7], increasing vulnerability to many addictive behaviors, including tobacco use [8,9,10]. Data from the United States (U.S.) indicate that 90% of *adult* smokers initiated smoking before 18 years of age [11], and data from China indicate that approximately 40% of *adolescent* smokers started smoking before 10 years of age [5,12]. Given the addictive nature of tobacco, it is of strategic significance to prevent adolescents from initiating and using tobacco products in order to curb the overall tobacco use epidemic.

Despite the need for tobacco use prevention among adolescents [13,14,15], basic data are lacking at the national level in China regarding secular trends in tobacco use among this high risk population. Only two national adolescent tobacco surveys have been conducted since the 1950s. One survey was conducted in 1988 by the China Centers for Disease Prevention and Control (China CDC, previously known as the Chinese Academy of Preventive Medicine, Beijing, China) and targeted participants 11–20 years old, an age range not commonly used in research [16]. The GYTS was conducted in 1999 by the U.S. Centers for Disease Control and Prevention (CDC) and China CDC. It targeted a segment of youth population that was 13–15 years of age [5]. As these two surveys were conducted 11 years apart, they are not very useful for describing time trends in detail.

A review of the published literature reveals a sizeable number of tobacco survey studies targeting adolescents at the local level from various parts of China, including Beijing [17,18], Shanghai [19], Wuhan [20], Hefei [21], Guangzhou [22], Xi’an [23], and Tibet [24]. Smoking rates reported from these studies varied dramatically. For example, the lifetime smoking rate (ever smoked in a lifetime) for males varied from 18.6% in Ningbo, Zhejiang Province (an eastern coastal city) [25] to 81.08% in Lhasa, Tibet (a western mountain city) [24]. These locally important data may contain information that is adequate for describing adolescent tobacco use at the national level using meta-analysis method [26]. However, no such studies were found in the published literature.

The purpose of this study was to fill in data gaps through meta-analysis to estimate prevalence rates of cigarette smoking among adolescents at the national level in China. Our analysis used smoking data available from high quality survey studies conducted during the 1981-2010 period. The ultimate goal is to provide basic data for decision-makers, tobacco researchers and controllers, and pediatric practitioners in China and across the globe for effective tobacco control.

## 2. Methods

### 2.1. Literature Search

The authors conducted the literature search, assisted by several graduate students. We targeted survey studies that were conducted during the 1981–2010 period and published in either Chinese or English. A set of 31 combinations of keywords were used in the literature search, including words reflecting study subjects (adolescent, adolescents, student, students, and youth), smoking behavior (cigarette, cigarettes, and smoking), and geographic locations (China and Chinese).

The search of the English literature was conducted on computers employing EndNote software (version x5, Thomson Reuters, San Francisco, CA, USA). Six English databases were searched, including Web of Science (1276 entries retrieved), PubMed (2027 entries retrieved), PsycINFO (522 entries retrieved), Cochrane (115 entries retrieved), Biological Abstracts (1207 entries retrieved), and Sociological Abstracts (181 entries retrieved). Our keyword searches yielded 5328 unduplicated entries from the six English language databases.

To search for the related literature in Chinese, a set of keywords similar to those for the English search was used. To maximize coverage, we selected the four largest, most comprehensive, and widely-used Chinese databases, including China National Knowledge Infrastructure (CNKI; 1218 entries retrieved), China Biomedical Literature Database (CBM; 1689 entries retrieved), Wan Fang (1044 entries retrieved), and VIP Chinese Journals Database (VIP; 485 entries retrieved). The Chinese keyword search yielded a total of 4436 unduplicated entries.

Considering the fact that not all studies published before the 1990s are electronically available, we manually searched over 30 key academic journals in Chinese and in English in the field of public health, school health, health education, preventive medicine, and behavioral medicine. Through the manual search, we identified and added eight articles.

### 2.2. Inclusion Criteria

To meet the goal of this study, we utilized the following criteria for paper selection: (1) The original study used cross-sectional surveys conducted (not published) in 1981–2010; (2) The target population contained: (a) middle school and/or high school students for school-based survey studies, or (b) adolescents within the age range of 12–17 for home-based survey studies; (3) the survey response rate was 90% or higher (response rates were high in most survey studies conducted in China); (4) descriptions were available regarding sampling, sample size, survey delivery, and data processing; (5) complete data were available on smoking behavior measures, including number of smokers and/or prevalence rates for both lifetime smoking (ever smoked in lifetime) and current smoking (smoked in the past 30 days); and (6) smoking data were reported by gender.

### 2.3. Paper Selection

**Figure 1:**
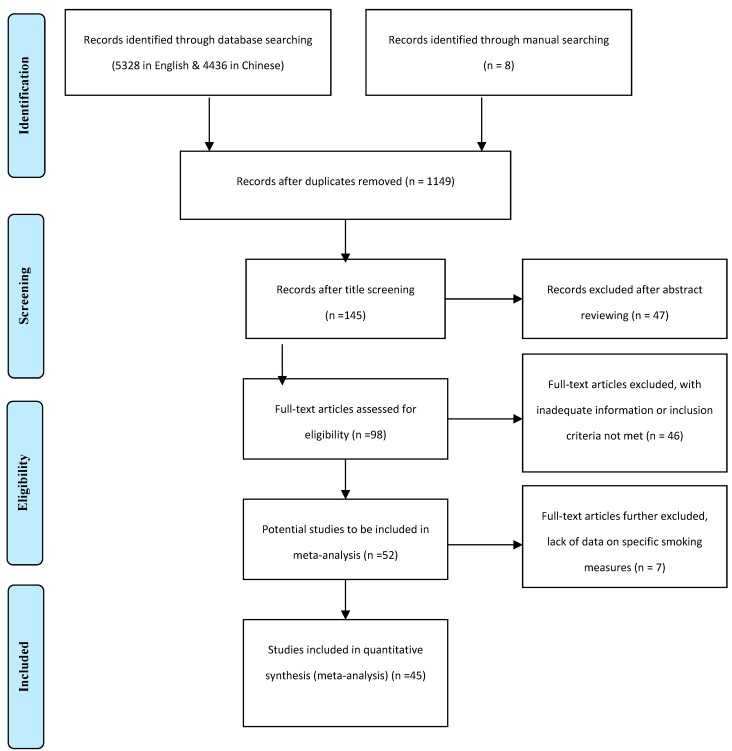
Flow diagram of paper selection for the meta-analysis.

The authors each independently assessed the located studies by carefully reviewing the abstracts and then judging them using the six inclusion criteria described above. They then worked together to decide which studies would be included and which excluded. In more than 90% of the cases, the decision was unanimous. When a decision could not be reached based on information included in the abstract, the full text of that paper was reviewed. Initial differences in paper selection between the authors occurred for five papers [24,25,27,28,29]. This was resolved through a thorough re-review followed by a group discussion. Of all the entries reviewed, 45 (16 in English and 29 in Chinese) met the criteria and were included. These selected papers are listed as supplementary information (Table S1). The search and selection process of these studies are summarized with the PRISMA flowchart (Figure 1).

### 2.4. Data Coding and Extracting

Data were extracted from the full text of the 45 selected papers. The following data were included: Type of survey (school-based or home-based), sampling method (random or convenience), school grade or age range of the survey participants, sample size, and definitions and measures of smoking behavior. Sample size and smoking measures were recorded by gender. The extracted data were manually entered into computers, and the computerized data were double-checked for accuracy.

### 2.5. Measurement of Smoking Behavior

To be consistent with published studies across the globe, we focused on two standard smoking behavior measures: *lifetime smoking (ever smoked)* and *current (30-day) smoking*. The former was conceptually defined as “having ever smoked in a lifetime” and the later was defined as “having smoked on at least one day in the past 30 days”. In the literature, one question form asked if a person “has ever smoked a cigarette in his/her life, including a few puffs” [30], and the other form asked if a person smoked “part or all of a cigarette in his/her life” [31]. We included these two forms to maximize the number of included studies for a more robust estimation.

Data from five studies were considered at the beginning and were later excluded. Two were GYTS surveys that contained data for China, but one report did not have smoking data by gender [5], and another report contained data by gender but without lifetime smoking data [32]. Efforts to obtain more detailed data breakdowns from the authors were not successful as of the publishing of this paper. Two other studies were the 2008–2010 multi-country Global Adult Tobacco Survey (GATS) [33] and the 2010 GATS [34]. China was included in these two surveys, but reported data did not include adolescents younger than 15 years of age, and the smoking rates for those aged 15–17 were included in the larger 15–24 age group, not suitable for use. In addition, the two GATS were household-based surveys, and smoking rates from such surveys have been shown to be less reliable [35]. The other two excluded studies were the 1984 China National Sampling Survey of Smoking [15] and the 1996 China National Prevalence Survey of Smoking [36]. These two adult surveys covered part of the adolescent population (aged 15–17), but they were included in the age group 15–24, and were not disaggregated for analysis.

### 2.6. Prevalence Rate Estimation by Meta-Analysis

Smoking prevalence rates were estimated using meta-analysis techniques [26] for each of the following six 5-year periods: 1981–1985, 1986–1990, 1991–1995, 1996–2000, 2001–2005, and 2006–2010. We selected five years as the interval to ensure an adequate number of studies in each period to allow for robust smoking rate estimates. With this design, all studies within a five-year period were used to estimate smoking rates for that period. Smoking rates were estimated using two different models: (1) the sample size weighted model and (2) the random effect model [26]. In the first model, the prevalence rates for each 5-year period were estimated as the total number of smokers over the total survey participants, as extracted from the selected studies conducted during the period. In the second model, each reported smoking rate during a five-year period was assumed to contain a between-studies variability *τ*^2^ around the grand mean and a within-study variability *σ_i_^2^* around the sample mean. The statistic *τ*^2^ was computed as the variance of smoking rates reported by all studies during the five-year period, and the *σ_i_^2^* for *i*th study was computed using the reported smoking rates and sample size of the study. The addition of the two (***τ***^2^ + *σ_i_^2^*) were used as weights to estimate the national rates [26].

The computing procedure for the meta-analysis was programmed using Microsoft Excel. In this report, we emphasized the results from the random effect modeling method for two reasons: (1) large variations in the reported smoking rates from different studies and (2) higher smoking rates for studies with large samples (a sample size-associated bias). The random effect method is more efficient than the sample-weighted method to deal with large variations and sample-size associated bias [26]. For comparison purposes, results from the two methods were tabulated together.

In contrast to meta-analyses that are based on a homogenous hypothesis in general to assess the effect of a treatment from multiple randomized trials, in our study we hypothesized that differences in the reported smoking rates by studies conducted in different regions within China differed from each other; therefore the differences were not primarily due to random error. Instead of conducting a heterogeneity test (such as Cochrane Q test) to assess homogeneity, we used the 95% confidence interval of the estimates to indicate the differences rather than random error [37].

### 2.7. Measures to Deal with Special Issues

To maximize the number of studies, data for individual study sites were treated as separate entries for two multi-site studies [38,39]. In addition, for the studies conducted in 1981–1990 in China, the current smoking rate was estimated based on the data from questions asking participants whether they smoked in the past 60 days, as recommended by the World Health Organization [40]. The reported smoking rates in these studies were not comparable to those estimated with data on smoking in the past 30 days. Reported studies among adolescents in China indicate that the ratio of past 30-day smoking over the past 60-day smoking = 0.91 for males and = 0.68 for females [41]. We applied these ratios to convert the past 60-day smoking to the past 30-day smoking.

## 3. Results and Discussion

### 3.1. Summary of the Included Studies

The Supplementary Material lists the 45 studies included in this analysis, of which 29 were in Chinese and 16 in English. There were four studies for 1981–1985, seven for 1986–1990, seven for 1991–1995, eight for 1996–2000, seven for 2001–2005, and 12 for 2006–2010. Of the 45 survey studies, 44 were school-based and one was community-based. Overall, these survey studies covered a total of 52 survey sites and 385,362 adolescents aged 12–17.

### 3.2. The Estimated Levels of and Time Trends in Lifetime Smoking

**Figure 2:**
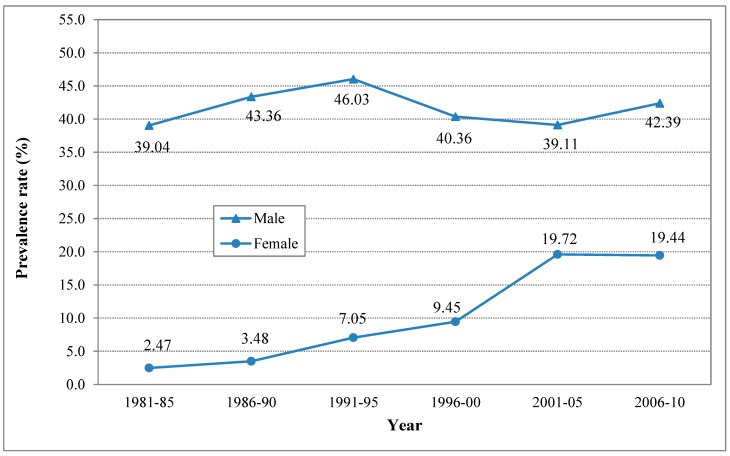
Estimated prevalence rate (%) of lifetime smoking (ever smoked) among adolescents in China, male and female, 1981–2010.

Figure 2 depicts the estimated prevalence rates of lifetime smoking in 1981–2010. The smoking rates for adolescent males varied within a narrow range between 39.04% (95% CI: 37.72, 40.36) and 46.03% (95% CI: 45.05, 47.01) during the period. Following an increasing trend from the 1981–1986 to 1991–1995, the lifetime smoking rates appear to have levelled off at around 40% after 1996.

Compared to males, the rates of lifetime smoking for females showed a different pattern. Starting at a relatively low level of 2.47% (95% CI: 1.75, 3.19) in 1981–1985, the prevalence rate increased progressively, and peaked at 19.72% (95% CI: 19.38, 19.86) in 2001–2005 before it levelled off slightly below 20%.

Results in Table 1 indicate that the time trends in the estimated rates of lifetime smoking using the two different methods were similar in most cases. However, as expected, relative to the random method, the rates estimated using the sample-weighted modeling method were significantly higher (exclusive 95% CI). This is likely because the sample-weight method is not efficient in handling the bias due to the inclusion of the studies with very large samples and high smoking rates in this analysis.

**Table 1:** Comparison of the estimated prevalence rates (95% CI) of lifetime smoking among adolescents in China using two different methods.

Gender	Time Period	Sample Weighted Method	Random Effect Method
	(Year)	Prevalence Rate (95% CI)	Prevalence Rate (95% CI)
**Male**	1980–1985	39.48 (38.16, 40.81)	39.04 (37.72, 40.36)
	1986–2000	39.71 (39.04, 40.37)	43.36 (42.69, 44.03)
	1991–1995	49.48 (48.50, 50.46)	46.03 (45.05, 47.01)
	1996–2000	42.24 (41.60, 42.88)	40.36 (39.73, 41.00)
	2001–2005	41.62 (41.32, 41.93)	39.11 (38.81, 39.41)
	2006–2010	42.27 (41.75, 42.80)	42.39 (41.87, 42.92)
**Female**	1980–1985	4.45 (3.49, 5.41)	2.47 (1.75, 3.19)
	1986–2000	4.10 (3.83, 4.38)	3.48 (3.23, 3.73)
	1991–1995	8.00 (7.43, 8.58)	7.05 (6.51, 7.60)
	1996–2000	11.17 (10.74, 11.61)	9.45 (9.04, 9.85)
	2001–2005	20.33 (20.08, 20.57)	19.72 (19.38, 19.86)
	2006–2010	20.31 (19.89, 20.73)	19.44 (19.02, 19.86)

### 3.3. The Estimated Levels of and Time Trends in Current (30-Day) Smoking

**Figure 3:**
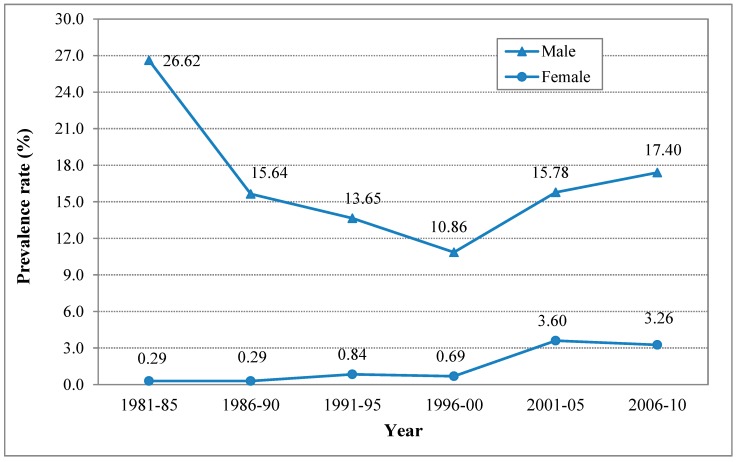
Estimated prevalence rate (%) of current smoking among adolescents in China, male and female, 1981–2010.

Figure 3 depicts the estimated prevalence rates and time pattern of current smoking among adolescents by gender. The male prevalence rate showed a “V” shaped pattern during the 1981–2010 period. Starting at the highest level of 26.62% (95% CI: 25.42, 27.81) in 1981–1985, the estimated smoking rates declined progressively before an increasing trend appeared after 2000.

The estimated current smoking rates for adolescent females showed a progressive increasing trend from 1981–1985 to 2006–2010. Starting at a low level of 0.29% (95% CI: 0.06, 0.51) in 1981–1985, the estimated smoking rates increased progressively before levelled off around 3.5% after 2001.

Likewise, results in Table 2 indicated that the time trends in the estimated rates of current smoking were similar regardless of estimation methods. In addition, the 95% CIs for the estimated rates of males in three periods (1981–1985, 1991–1995 and 1996–2000) and of females in two periods (1981–1985 and 1991–1995) were mutually exclusive, indicating no significant difference between the two estimation method.

**Table 2:** Comparison of the estimated prevalence rates (95% CI) of current smoking among adolescents in China using two different methods.

Gender	Time Period	Sample Weighted Method	Random Effect Method
	(Year)	Prevalence Rate (95% CI)	Prevalence Rate (95% CI)
**Male**	1981–1985	26.95 (25.75, 28.15)	26.62 (25.42, 27.81)
	1986–1990	12.54 (12.04, 13.04)	15.64 (15.09, 16.19)
	1991–1995	14.04 (13.36, 14.72)	13.65 (12.97, 14.32)
	1996–2000	10.35 (9.96, 10.75)	10.86 (10.46, 11.26)
	2001–2005	19.40 (19.15, 19.64)	15.78 (15.56, 16.01)
	2006–2010	19.51 (19.09, 19.93)	17.40 (17.00, 17.80)
**Female**	1981–1985	0.29 (0.06, 0.51)	0.29 (0.06, 0.51)
	1986–2000	0.47 (0.37, 0.58)	0.29 (0.20, 0.37)
	1991–1995	0.89 (0.68, 1.09)	0.84 (0.64, 1.04)
	1996–2000	1.25 (1.10, 1.41)	0.69 (0.58, 0.80)
	2001–2005	4.41 (4.29, 4.54)	3.60 (3.49, 3.72)
	2006–2010	4.89 (4.66, 5.12)	3.26 (3.08, 3.45)

## 4. Discussion

Tobacco control has been recognized as the “most urgent and immediate priority” to reduce non-communicable diseases [42]. To the best of our knowledge, this is the first meta-analysis reporting the estimates of national prevalence rates of Chinese adolescent tobacco use using data from published studies conducted in various parts of China at local level. Findings of this study extended the newly published prevalence data on adults smoking in China from the GATS [32] and will increase the value of the newly released data from the 2014 GYTS (http://www.wpro.who.int/mediacentre/releases/2014 /20140618/en/). These data are essential for public health researchers, practitioners, and decision-makers to understand the situation of adolescent tobacco use in China and to plan for future tobacco research and control.

### 4.1. A Growing Number of Adolescent Smokers in China

The first and most striking finding of this study is that there has been a rapid increase in smoking among adolescents in China since 1996–2000. According to the estimated rates of lifetime smoking in 2006–2010 and the 2010 China Census data, each year a total of 32.85 million youth (95% CI: 32.37, 33.36 million), including 23.31 million males (95% CI: 23.04, 23.59) and 9.53 million females (95% CI: 9.33, 9.77) start (ever tried) smoking. This means that approximately 90,000 Chinese adolescents start trying cigarettes every day. Furthermore, the smoking initiation rate for adolescent females showed a rapid and progressive increase trend since the 1980s.

The prevalence of current smoking is also of concern. According to the estimated rates and the same adolescent population data from the 2010 Census, a total of 11.18 million (95% CI: 10.87, 12.06) adolescents are currently smoking cigarettes, including 9.57 million (95% CI: 9.35, 10.34) males and 1.62 million (95% CI: 1.52, 1.72) females who are current smokers. The current smoking rate of 17.40% among Chinese adolescent males in 2006–2010 was close to 19.5%, the rates for U.S. adolescents in 2009 [30]. Although the current smoking rate for adolescent females is relatively low compared to their male counterparts, it has increased progressively since the 1980s. The high and growing levels of tobacco use among adolescents could predict a growing future burden from tobacco-related diseases in China, including cancers, cardiovascular diseases, and mental and behavioral health problems.

### 4.2. Changing Gender Pattern in Cigarette Smoking

As has been observed in China and many other Asian countries, large and persistent gender differences in tobacco use have traditionally been considered normative [14,16]. However, this traditional perception may soon be out of date. Data from our analysis indicate rapid reductions in the gender gap from 1981 to 2006 for lifetime smoking and from 1981 to 2001 for current smoking. The difference in the lifetime smoking between males and females was over 15 times (39.04% *vs.* 2.47%) in 1981–1985; this difference declined to less than 2 (39.11% *vs.* 19.72%) in 2001-05. The gender gap reduction was also striking for current smoking. The changing gender pattern revealed in this meta-analysis was consistent with the findings of increased smoking among young female adults from the more recently published GATS data [33,34].

A number of factors may explain the increasing trends in female adolescent smoking in China. Along with the economic globalization and urbanization, tobacco industries (including transnational companies in China) have increased their effort to entice young women to use tobacco. Survey data indicate that Chinese adolescents are highly receptive to pro-tobacco media [40]. Reported studies also show that female smokers are portrayed as “fashionable”, “elegant”, and “cool” by the tobacco industry, and tobacco products are promoted as needed for cognitive work, mood regulation, and weight control; all of which are effective in convincing young women to smoke [43,44,45].

Further evidence supporting the effectiveness of targeted marketing by tobacco industries in China is a more recent finding that many Chinese females now agree that “smoking looks cool”, “smoking is good for social networking”, and “smoking makes people feel comfortable” [46]. In the U.S., it took approximately two decades for female adolescents to reach their male counterparts in levels of cigarette smoking in 1975 [47]. If no specific measures are taken, the gender difference in tobacco use among Chinese adolescents could soon disappear.

### 4.3. Implications for Tobacco Control

Tobacco use is a global epidemic, and preventing young people from smoking represents the best strategy against the epidemic [40,41,42]. Tobacco use costs China $29 billion dollars per year [48], indicating an urgent need for tobacco control. Successful experiences across the globe have repeatedly demonstrated that tobacco is one of the most preventable causes of increased morbidities and mortalities [10,40,47,48,49]. If no additional measures are taken, many tobacco-related diseases will increase, particularly lung cancer, high blood pressure, stroke and other cardiovascular diseases, mental and behavioral problems. To successfully curb the increasing trends in tobacco use among Chinese adolescents, comprehensive strategies must be adapted, including quitting clinics to assist addicted smokers stop smoking with smoking cessation, school-based interventions, social marketing, tobacco taxation, and other policy and legal measures to prevent initiation and use of tobacco products among adolescents.

There are limitations to this study. Despite the other strengths of this study, results from meta-analysis can only serve as a complementary measure and cannot replace data from regular sampling surveys at the national level in order to better describe patterns and changes in adolescent tobacco use. Also, publication biases in the estimated rates could not be totally ruled out given the reported studies available for this analysis. For example, the lower rates of lifetime and current smoking in 1996–2000 for males could be at least in part due to the fewer number of studies included in this meta-analysis from the northeast and southwest regions of the country, as these regions are well known for having higher smoking rates [5]. The limited number of studies for several periods prevented this study from systematically assessing the impact of geographic differences through meta-regression analysis. Therefore, caution is needed when interpreting the results. Additionally, data used for this study are primarily from school-based survey studies. Smoking behaviors among adolescents who did not attend school are not represented. Lastly, the age range of middle and high school students in the reported studies was likely to vary outside of the bounds of the age-group 12–17, which may impact the accuracy of the estimated smoking rates. Despite the limitations, the findings of this study are of timely significance, and support the urgent need for greater clinical and public health efforts to address the high smoking levels among adolescents in China, and to curb increasing trends.

## 5. Conclusions

The study analyzed the data with the lifetime and current smoking rates for Chinese adolescents in 1981–2010 through a meta-analysis of 45 different studies. Results showed high levels of male adolescent smoking and rapidly increasing trends in female adolescent smoking in China. This result underscores the urgency for strengthening tobacco control strategies in China. Attention should be also devoted to the rapidly growing number of adolescent female smokers in China.

## Supplementary Files

Supplementary File 1
