# Supplementary material for: A Meta-Analysis of Cigarette Smoking Prevalence among Adolescents in China: 1981–2010
Source: Int J Environ Res Public Health. 2015 Apr 27;12(5):4617–30. doi: 10.3390/ijerph120504617 (PMC4454929; doi:10.3390/ijerph120504617)
Supplement: Supplementary File 1 [file ijerph-12-04617-s001.pdf]

A Meta-Analysis of Cigarette Smoking Prevalence among Adolescents in China: 1981–2010

Table S1. Studies on smoking of adolescents in China during 1981–2010.

Study	Study Year	Location	Grades	Male Sample	Female Sample	Lifetime Prevalence for Male	Lifetime Prevalence for Female	Current Prevalence for Male	Current Prevalence for Female
1981–1985									
[1] Ye, G.S.; Lin, W.S. (1982)	1981	Beijing	7–11	1396	1394	24.9	ND	19.03	0.22
[2] Liu, X. (1986)	1985	Henan	7–12	1314	800	46.72	9.0	28.89	0.41
[3] Wang, T.; Liu, M. (1988)	1985	Anshan	7–12	1213	ND	39.9	ND	26.36	0
[4] The department of school health, (1986)	1983	Gansu	7–12	1326	974	47.29	0.72	33.91	ND
1986–1990									
[5] Hu, J.F.; *et al.* (1990)	1988	Harbin	7–12	1786	1863	33.48	3.28	13.84	1.18
[6] Shu, A.; *et al.* (1993)	1988	Beijing	7–12	2881	2437	36.7	2.3	ND	ND
[7] Niu, S.; *et al.* (1995)	1990	Fuzhou/Xiamen/Shenyang/Chengdu	7–12	7771	7337	38.31	4.43	6.56	0.11
[8] Liu, K.; *et al.* (1991)	1989	Shenyang	7–12	1181	1138	35.3	0.9	ND	ND
[9] Zhu, B.P.; *et al.* (1992)	1988	Beijing	7–9	4241	4196	34.38	3.9	13.18	0.77
[10] Wang, S.Q.; *et al.* (1994)	1988	Beijing	10–12	1923	1900	46.0	5.5	18.06	0.83
[11] Zhang, M.; *et al.* (1990)	1987	lhasa	7–12	1121	1448	81.08	7.8	40.09	0.06
1991–1995									
[12] Liu, X. *et al.* (2003)	1993	Shandong	7–12	816	544	31	3.7	6.38	0.50
[13] Osaki, Y; *et al.* (1999)	1991	Jiangxi	7–12	6393	5212	52.3	7.1	11.7	0.6
[14] Crowe, J.W.; *et al.* (1994)	1991	Beijing	7–8	201	192	24.3	5.6	9.9	0
[15] Liu, G. R. (1997)	1993	Shandong	10–12	163	282	54.69	1.52	14.84	0
[16] Zhou, C.Q.; *et al.* (1993)	1991	Nanning	10–12	1250	1166	54.72	12.26	29.06	2.32
[17] Bi, P.; *et al.* (1995)	1993	Hefei	7–12	830	784	47.23	11.48	18.62	0.49
[18] Fang, X.; *et al.* (1996)	1991	Beijing	6, 8, 10	322	344	38.82	12.78	11.86	1.88

Table S1. *Cont.*

Study	Study Year	Location	Grades	Male Sample	Female Sample	Lifetime Prevalence for Male	Lifetime Prevalence for Female	Current Prevalence for Male	Current Prevalence for Female
1996–2000									
[19] Yang, G.; *et al*. (2004)	1998	national	7–12	9151	8061	47.22	11.59	7.54	0.25
[20] Unger, J. B.; *et al*. (2001)	1998	Wuhan	7–9	3707	3287	47	18	16	3
[21] Hesketh, T.; *et al*. (2001)	1998	Zhejiang	7–12	3470	3204	26.49	5.29	1.27	0.25
[22] Zhang, L.;*et al*. (2000)	1996	Henan	7–12	1668	1614	30.33	5.34	13.50	0.79
[23] Liu, Z.; *et al*. (2001)		Guizhou	11	1416	1068	52.4	8.9	19.3	0.7
[24] Zhou, D.; *et al*. (2000)		Suzhou	7–12	407	372	22.4	1.9	8.11	0.28
[25] Tao, F.B.; *et al*. (2006)	1998	Hefei	7–12	1224	820	46.3	16.5	4.8	0.2
[26] Zhang, H.; *et al*. (1999)	1998	Yunnan	7–12	1844	1751	42.25	13.42	24.54	5.91
2001–2005									
[27] Anderson Johnson, C; *et al*. (2006)	2002	Harbin	7–12	761	820	41.26	22.93	12.88	5.98
		Shenyang	7–12	881	891	40.18	20.53	12.49	3.59
		Wuhan	7–12	861	985	42.74	27.01	12.08	2.94
		Chengdu	7–12	877	821	46.98	32.03	14.82	6.21
		Kunming	7–12	879	862	56.31	27.38	21.5	5.57
		Hangzhou	7–12	832	782	29.09	21.1	5.77	4.09
		Qingdao	7–12	795	894	26.29	15.44	7.42	2.68
[28] Li, X.; *et al*. (2010)	2001	Nanjing	7–9	495	487	31	16	21	8
[29] Li, J.; *et al*. (2005)	2002	Shanghai	7–12	1097	1107	27.8	10.84	10.57	1.9
[30] Wang, P.; *et al*. (2007)	2004	Liuzhou	7–12	1565	1841	49.9	26.7	20.2	4.8
[31] Wen, X., *et al*. (2008)	2004	Guangzhou	7–9	1330	1294	26.5	7.2	8.02	0.44
[32] Tao, F.B.; *et al*. (2006)	2003	Hefei	7–12	1143	877	48.9	19.8	19.9	1.8
[33] Ji, C.; *et al*. (2009)	2005	National	7–12	88,808	93,024	41.9	20.3	20.1	4.5

Table S1. *Cont.*

Study	Study Year	Location	Grades	Male Sample	Female Sample	Lifetime Prevalence for Male	Lifetime Prevalence for Female	Current Prevalence for Male	Current Prevalence for Female
2006–2010									
[34] Qing, Y.; *et al*. (2011)	2009	Anhui	7–12	1709	1522	41.49	17.67	16.2	4.3
[35] Zhu, Y.; *et al*. (2011)	2009	Ningbo	7–8	2452	2257	18.6	7.09	1.94	0.54
[36] Xu, Z.; *et al*. (2010)	2006	Zhangjiagan	7–12	2535	2362	24.7	9.8	8	1.3
[37] Shi, X.; *et al*. (2010)	2008	Nanning	7–12	1486	1502	46.6	25.5	22	5.8
[38] Yan, J.; *et al*. (2011)	2010	Beijing	7–12	617	582	41.2	17.5	24.3	6.2
[39] Wang, S.;*et al*. (2011)	2010	Beijing	7–12	655	791	51.9	17.2	24.4	2.3
[40] Hu, P.; *et al*. (2011)	2008	Beijing	7–12	8899	9331	35.7	21.8	18.9	7
		Yunnan	7–12	4015	4641	62.4	30.9	36.4	10.2
[41] Fang, H.;*et al*. (2008)	2006	Xi'an	7–12	568	584	41.7	19.5	11.6	1.7
[42] Yang, B.; *et al*. (2011)	2008	Henan	10–12	3283	3861	61.7	24.8	36.37	4.27
[43] Gao, B.; *et al*. (2010)	2008	Henan	7–9	1254	1112	35.5	13.6	14.9	3.3
[44] Zhang, J.;*et al*. (2011)	2010	Beijing	7–12	940	734	53.3	20.7	14.7	1.4
[45] Wang, H.; *et al*. (2008)	2007	Zhejiang	7–12	5913	5725	42.99	17.19	13.58	1.99

References

1. Ye, G.S.; Lin, W.S. Cigarette smoking among Beijing high schoolers. *Chin. Med. J.* **1982**, *95*, 95–100.
2. Liu, X. Survey on the status of smoking among 2144 middle school students in Nanzhao county. *Sch. Health* **1986**, *7*, 28–30. (In Chinese)
3. Wang, T.; Liu, M. Survey on cigarrete smoking situation of middle school students in Anshan city. *Sch. Health* **1988**, *9*, 6–7. (in Chinese)
4. The department of school health, sanitation antiepidemic station in Gansu province. The analysis of smoking habit among 2300 middle school students in urban and rural aeras, Guansu. *Sch. Health* **1986**, *7*, 25–27.
5. Hu, J.F.; Liu, R.Z.; Zhang, H.L.; Xu, X.F.; Li, K.; Yang, R.Z.; Li, S.X.; Zhang, Z.T. A survey of cigarette smoking among middle school students in 1988. *Public Health* **1990**, *104*, 345–351.
6. Shu, A.; Zhou, Y.; Wen, X.; Sun, F.; Li, B.; Qi, H.; Zhang,Y.; Du, Q.; Liu, Z. Sample survey on smoking status of middle school students in Chaoyang district ,Beijing. *J.Cardiovasc. Pulm. Dis.* **1993**, *12*, 119–120.
7. Niu, S.; Guan, N.; Tian,B.; Guan, J.; Zhang, D.; Liu, Y.; Lu, Z.; Guo,H.; Lu, B.; Guo, B. The research on smoking status among 30 thousand middle school and university students. *J. Hyg. Res.* **1995**, *24*, 15–18.
8. Liu, K.; Li, G.; Sun, W.;Ye, L.;Zhao, X. Investigation of smoking act and influencing factors in genernal middle school students. *Chin. J. Public Health Eng.* **1991**, *10*, 4–6.
9. Zhu, B.P.; Liu, M.; Wang, S.Q.; He, G.Q.; Chen, D.H.; Shi, J.H.; Shang, J.Z. Cigarette smoking among junior high school students in Beijing, China, 1988. *Int. J. Epidemiol.* **1992**, *21*, 854–861.
10. Wang, S.Q.; Yu, J.J.; Zhu, B.P.; Liu, M.; He, G.Q. Cigarette smoking and its risk factors among senior high school students in Beijing,China, 1988. *Tob. Control* **1994**, *3*, 107–114.
11. Zhang, M.; Liang, Z.; Zhou, Y.; Yang, N. Survey on the status of smoking among 2569 middle school students of Thibetan in Lhasa city. *Chin. Sch. Dr.* **1990**, *4*, 20–22.
12. Liu, X. Cigarette smoking, life stress, and behavioral problems in Chinese adolescents. *J. Adolesc. Health* **2003**, *33,* 189–192.
13. Osaki, Y.; Minowa, M.; Mei, J. A comparison of correlates of cigarette smoking behavior between Jiangxi province, China and Japanese high school students. *J. Epidemiol.* **1999**, *9*, 254–260.
14. Crowe, J.W.; Torabi, M.R.; Nakornkhet, N. Cross-cultural study of samples of adolescents' attitudes, knowledge, and behaviors related to smoking. *Psychol. Rep.* **1994**, *75*, 1155–1161.
15. Liu, G.R. An investigation of adolescent health from China. *J. Adolesc. Health* **1997**, *20*, 306–308.
16. Zhou, C.; Chen, H. Survey of smoking condition among senior high school students in urban, Nanning city. *Chin. Sch. Dr.* **1993**, *7*, 65–68.
17. Bi, P.; Sun, Y. The epidemiological survey on smoking behavior and initiating factors of middle school students in Hefei. *Chin. Sch. Health* **1995**, *16*, 40–41.
18. Fang, X.; Li, X. Dong, Q. Study on smoking of adolescent and related factors. *Chin. Mental Health* **1996**, *10*, 77–80.
19. Yang, G.; Ma, J.; Chen, A.P.; Brown, S.; Taylor, C.E.; Samet, J.M. Smoking among adolescents in China: 1998 survey findings. *Int. J. Epidemiol.* **2004**, *33*, 1103–1110.

20. Unger, J.B.; Li, Y.; Chen, X.; Xia, J.; Sun, A.Z.; Guo, Q.; Tan, S.; Gong, J.; Sun, P.; Liu, C.H.; Chou, C.P.; Zheng, H.; Anderson Johnson, C. Adolescent smoking in Wuhan, China: Baseline data from the Wuhan Smoking Prevention Trial. *Amer. J. Prev. Med.***2001**, *21*, 162–169.
21. Hesketh, T.; Ding, Q.J.; Tomkins, A. Smoking among youths in China. *Amer. J. Public Health* **2001**, *91*, 1653–1655.
22. Zhang, L.; Wang, W.; Zhao, Q.; Vartiainen, E. Psychosocial predictors of smoking among secondary school students in Henan, China. *Health Educ. Res.* **2000**, *15*, 415–422.
23. Liu, Z.; Zhou, W.; Lian, Z.; Mu, Y.; Cai, Z.; Cao, J. The use of psychoactive substances among adolescent students in an area in the south-west of China. *Addiction* **2001**, *96*, 247–250.
24. Zhou, D.; Tang, H. Li, S. The analysis of life misconduct and related factors of middle school students. *Chin. J. Sch. Dr.* **2000**, *14*, 96–97.
25. Tao, F.B.; Huang, K.; Gao, M.; Su, P.Y. Smoking and subjective life qualities in middle school students. *Chin. J. Epidemiol.* **2006**, *27*, 132–136.
26. Zhang, H.; Guo, S.; Zhao, H. The epidemiological survey od smoking and drinking abuse among 3595 adolescents. *Chin. Sch. Dr.* **1999**, *13*, 86–89.
27. Anderson, J.C.; Palmer, P.H.; Chou, C.P.; Pang, Z.; Zhou, D.; Dong. L.; Xiang, H.; Yang, P.; Xu, H.; Wang,J.; *et al*. Tobacco use among youth and adults in Mainland China: The China seven cities study. *Public Health* **2006**, *120*, 1156–1169.
28. Li, X.; Mao, R.; Bonita, S.; Zhao, Q. Parental, behavioral, and psychological factors associated with cigarette smoking among secondary school students in Nanjing, China. *J. Child Fam. Stud.* **2010**, *19*, 308–317.
29. Li, J.; Li, X.; Peng, N. Analysis of smoking behavior and its related factors among adolescents in certain districts of Shanghai. *Chin. J. Sch. Dr*. **2005**, *19*, 111–114.
30. Wang, P.; Zeng, X.L.; Yan, Z.L.; Yang, B.H. The status of smoking among adolescents in Liuzhou. *Chin. J. Dis. Control Prev.* **2007**, *11*, 112–114.
31. Wen, X.; Chen, W.; Qian, Z.; Muscat, J.E.; Lu, C.; Ling, W. Differences in students' smoking-related knowledge, attitudes, and behaviors among public, factory, and private secondary schools in Guangzhou, China*. J. Sch. Health* **2008**, *78*, 46–53.
32. Tao, F.B.; Huang, K.; Gao, M.; Su, P.Y. Smoking and subjective life qualities in middle school students. *Chin. J. Epidemiol.* **2006**, *27*, 132–136.
33. Ji, C.; Chen, T.; Song, Y.; Hu, P.; Xing, Y.; Zhang, L. Smoking status of high school and college students in China. *Chin. J. Sch. Health* **2009**, *30*, 109–111.
34. Qing, Y.; Termsirikulchai, L.; Vatanasomboon, P.; Sujirarat, D.; Tanasugarn, C.; Kengganpanich, M. Factors related to tobacco use among middle school students in China. *Southeast Asian J. Trop. Med. Public Health* **2011**, *42*, 1249–1261.
35. Zhu, Y.; Zhang, T.; Wang, X.; Gao, H.; Chen, J.; Shen Y. Survey on smoking behavior among junior high school students in 6 district of Ningbo. *Chin. J. Prev. Med.* **2011**, *45*, 464–466.
36. Xu, Z.; Zhu, H.; Yu, Y. Survey on the influencing factors of smoking and drinking for middle school students in Zhangjiagang. *Chin. J. Sch. Health* **2010**, *31*, 469–471.
37. Shi, X.; Lu, J.; Liang, H.; Liu, X.; Su, P.; Long, X.; Lu, S. Epidemiological study of smoking among adolescents in Nanning. *Chin. J. Sch. Health* **2010**, *31*, 1180–1182.

38. Yan, J.; Zhang, T. Smoking among middle school students in Huairou district of Beijing city. *Occup. Health* **2011**, *27*, 1085–1087.
39. Wang, S.; Zhang, Z. Tobacco use status and KAP survey among 1446 middle school students in a district of Beijing. *Chin. J. Sch. Health* **2011**, *32*, 646–651.
40. Hu, P.; Ji, C.; Song, Y. Comparison of cigarette smoking among adolescents in Beijing and Yunnan province: Data from Chinese national youth risk behaviors surveillance. *Chin. J. Sch. Health* **2011**, *32*, 394–396.
41. Fang, H.; Ding, Y.; Li, N. Survey on adolescents' knowledge, attitude, and behavior about smoking in Xi'an. *Chin. J. Health Educ.* **2008**, *24*, 19–20, 26.
42. Yang, B.; He, J.; Zhong, Y. Survey on substance abuse among senior high school students in four cities of Heinan province. *Henan J. Prev. Med.* **2011**, *22*, 187–188, 192.
43. Gao, B.; Zhong, Y.; Yang, B. Survey on cigarette smoking and alcohol drinking behavior among urban junior school students in 2008, Henan province. *Henan J. Prev. Med.* **2010**, *21*, 191–192. (In Chinese)
44. Zhang, J.; Cui, Y.; Huang, Y.; Liu,Y.; Liu, X.; Wang, J.; Ten, S. Smoking related knowledge,attitudes and behaviors among middle school students in Changping, Beijing, 2010. *Chin. Prev. Med.* **2011**, *12*, 695–699.
45. Wang, H.; Yu, M.; Hu, R.Y.; Wang, L.X.; Gong, W.W. Survey of smoking middle school students in Zhejiang. *Dis. Surveill.* **2008**, *23*,114–116.

© 2015 by the authors; licensee MDPI, Basel, Switzerland. This article is an open access article distributed under the terms and conditions of the Creative Commons Attribution license (http://creativecommons.org/licenses/by/4.0/).
